# Pure single‐port retzius‐sparing robot‐assisted radical prostatectomy with the da Vinci SP: Initial experience and technique description

**DOI:** 10.1002/bco2.131

**Published:** 2022-01-12

**Authors:** Periklis Koukourikis, Ali Abdullah Alqahtani, Woong Kyu Han, Koon Ho Rha

**Affiliations:** ^1^ Department of Urology, Urological Science Institute Yonsei University College of Medicine Seoul South Korea; ^2^ Second Department of Urology, School of Medicine Aristotle University of Thessaloniki Thessaloniki Greece

**Keywords:** prostate cancer, prostatectomy, robotics, single‐access surgery, single port

## Abstract

**Objective:**

To assess the feasibility and safety of pure single‐port (SP) retzius‐sparing robot‐assisted radical prostatectomy (RARP) using the da Vinci SP and describe the technique.

**Materials and Methods:**

From August 2020 to November 2020, data of 10 consecutive patients with localized prostate cancer, who underwent SP retzius‐sparing RARP, were prospectively collected. Patients demographics, intraoperative variables, postoperative complications, early oncological, and functional outcomes were assessed.

**Results:**

The patients were aged 46–73 years with a body mass index between 20.3 and 27.4 kg/m^2^. Prostate volumes ranged from 15 to 47.2 ml, with a median (interquartile range, IQR) PSA level of 7.4 (6.2–9.1) ng/ml. All surgeries were successfully completed without conversion. The median (IQR) operative and console time were 106 (101–109) min and 65 (63–68) min, respectively. The median (IQR) blood loss was 125 (50–150) ml, and one Clavien–Dindo grade I complication occurred. At 3 months, nine patients had undetectable PSA levels and all patients were continent.

**Conclusions:**

Pure SP retzius‐sparing RARP could be safely performed using the da Vinci SP system, with acceptable surgical times and minimal complications. Future research will evaluate the advantages of this technique over the standard multiport robotic surgery.

## 1 INTRODUCTION

Since the introduction of robotics in urology and initial description of robot‐assisted radical prostatectomy (RARP) in 2000, urologists have been seeking modifications of the procedure in an effort to minimize the disruption of the periprostatic anatomy, achieve optimal functional outcomes, and reduce morbidity.^1^, ^2^ Under this goal, Galfano et al.^3^ first described the Bocciardi or retzius‐sparing approach for radical prostatectomy in 2010. During this approach, the entire procedure is performed through the pouch of Douglas while the anterior supportive structures contained in the retzius space are preserved. Two randomized controlled trials showed the superiority of retzius‐sparing surgery in early continence recovery, and the technique adoption has been largely distributed among urologists around the globe.^4^, ^5^, ^6^

The da Vinci single‐port (SP) Surgical System (Intuitive Surgical, Sunnyvale, CA, USA) is the first purpose‐built SP surgical platform; the system utilizes a 12‐mm × 10‐mm articulating camera and three 6‐mm double‐jointed robotic instruments, all inserted through a 25‐mm multichannel port.^7^ Since the approval of the da Vinci SP by the FDA in 2018, new ways of performing urologic surgery have been explored.^8^

In the present study, we demonstrate the technical feasibility of pure SP retzius‐sparing RARP using the da Vinci SP; we report on surgical technique, initial experience, and short‐term outcomes of our technique.

## 2 PATIENTS AND METHODS

### 2.1 Study population

From August 2020 to November 2020, data of 10 consecutive patients who underwent SP retzius‐sparing RARP, by a single surgeon (KHR), for clinically localized prostate cancer were prospectively collected in an institutional review board‐approved database. All patients underwent multiparametric magnetic resonance imaging (mpMRI) of the pelvis and bone scan for staging. Exclusion criteria for enrolment were preoperative evidence of extracapsular or metastatic disease. Patients with previous prostate and/or abdominal surgery were also excluded.

### 2.2 Outcomes measures

Baseline characteristics and clinical data of the patients were collected, including age, body mass index (BMI), Charlson comorbidity index (CCI), American Association of Anesthesiologist (ASA) score, prebiopsy PSA level, biopsy International Society of Urological Pathology (ISUP) grade, and clinical stage. Preoperative urinary and sexual function were assessed using the International Consultation on Incontinence Questionnaire–Urinary Incontinence Short‐Form questionnaire and Sexual Health Index for Men (SHIM) score, respectively.^9^, ^10^ The collected intraoperative data were docking time, console time, urethrovesical anastomosis time, total operating time, estimated blood loss (EBL), complications, blood transfusion, and conversion. Postoperatively, hospitalization time, catheterization time, surgical margins status, pathology ISUP grade, pathology clinical stage, and complications within 30 days, according to Clavien–Dindo classification,^11^ were recorded. Continence was assessed the day of catheter removal, at 1 and 3 months after surgery. Continence was defined as use of no pads or one safety liner per day.^12^ PSA was measured at 1 and 3 months after surgery, and biochemical recurrence (BCR) was defined as two consecutive elevations of serum PSA > 0.2 ng/ml, postoperatively.

The primary outcomes of this study were feasibility of the technique in terms of conversion to anterior approach or multiport surgery and patient safety in terms of intraoperative and postoperative complications. Secondary outcomes that were assessed were perioperative, early oncological, and functional outcomes.

### 2.3 Surgical technique

**FIGURE 1:**
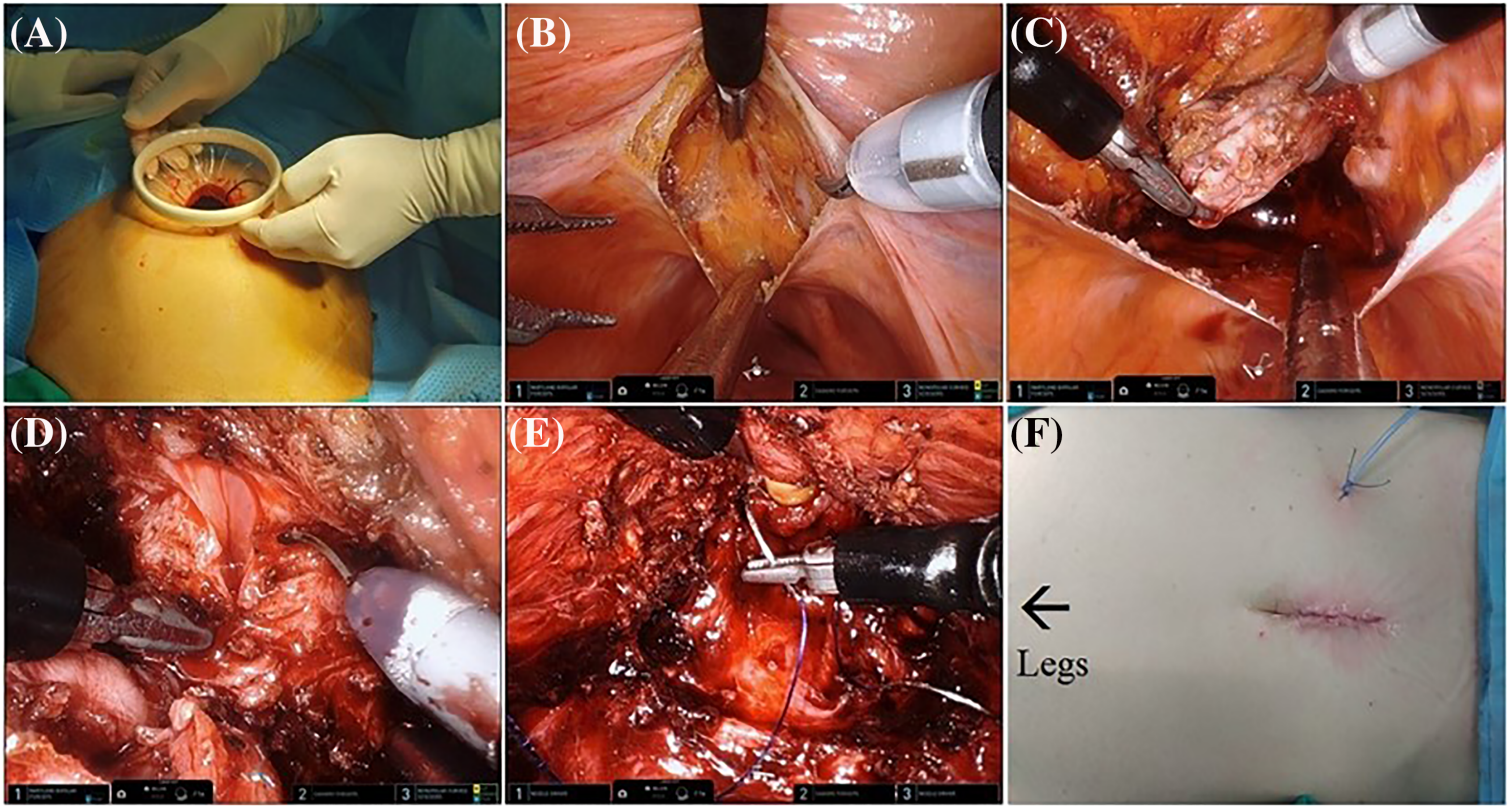
Outline of still images of pure SP retzius‐sparing RARP: (A) Incision and Alexis wound retractor placement, (B) peritoneal incision at the pouch of Douglas, (C) seminal vesicle dissection, (D) bladder neck dissection, (E) urethrovesical anastomosis, and (F) final incision and drain. SP, single‐port; RARP, robot‐assisted radical prostatectomy

Patient positioning for SP retzius‐sparing RARP is largely similar to our multiport approach; the patients are placed in lithotomy position, with legs in stirrups, and secured to the operating table. The abdomen is draped in the usual sterile manner. A 35‐ to 40‐mm supraumbilical midline incision is performed, and the peritoneal cavity is accessed to place the Alexis wound retractor (Applied Medical, Rancho Santa Margarita, CA, USA) (Figure 1A). We utilize a GelPOINT advanced access platform (Applied Medical, Rancho Santa Margarita, CA, USA) to place the 25‐mm SP port and an 12‐mm assistant port; both are placed to the gel‐seal cap. The gel‐seal cap is attached to the wound retractor, and the abdomen is insufflated at 12 mmHg.

The patient is placed in 20° Trendelenburg position, and the SP robot is docked. The configuration of the instruments is as follows: the articulating camera at 6 o'clock of the multichannel port and intraoperative field, the monopolar scissors in the right‐hand position in instrument #1; bipolar Maryland in the left‐hand position in instrument arm #3, and Cadiere forceps at 12 o'clock position in instrument arm #2.

Any adhesions of the sigmoid to left lateral abdominal wall are freed, and the bowels are moved cranially to exposure the pouch of Douglas. An incision is performed in parallel to the superficial transverse vein in the posterior peritoneum, slightly above the level of the vas deferens (Figure 1B). The vasa are ligated bilaterally; the seminal vesicles are retracted upwards by the Cardiere forceps and dissected from the surrounding tissues (Figure 1C). The Denonvilliers' fascia is incised, and the posterior plane is developed until reaching the prostate‐urethral junction. The prostatic pedicles are ligated and divided bilaterally. A combination of sharp and blunt dissection is applied to the lateral prostate aspect until the apex and deep vein complex are seen. Then, the bladder neck is dissected sharply recognizing the circumferential detrusor muscle fibers (Figure 1D). The anterior dissection is continued, sparing the detrusor apron and pubovesical complex, towards the urethra which is sharply transected. The specimen is placed in a specimen bag and guided to the right upper quadrant by the assistant.

The Maryland forceps and the monopolar scissors are replaced by two needle holders. For the urethrovesical anastomosis two 3‐0 absorbable barbed sutures are used, one 23 cm and one 15 cm, on a SH‐needle (Monofix, Samyang Biopharm, Korea). The anterior bladder neck is anastomosed to the anterior urethra starting from 12 o'clock to 3 o'clock. The same procedure is repeated from 11 o'clock to 9 o'clock using the 15 cm long suture. The urethral catheter is advanced into the bladder, and the anastomosis is concluded when the two sutures meet at 6 o'clock and are tied together (Figure 1E). A new 14‐Fr silicone catheter is inserted, and the water tightness of the anastomosis is checked installing 150 ml of saline.

The prostatectomy bed is packed with absorbable hemostatic agents and tissue glue is applied. The peritoneal incision is closed in a continuous manner with 3‐0 absorbable barbed suture. The robot is undocked and the specimen is retrieved. A 10‐Fr Jackson Pratt drain is placed through a stamp incision. The fascia and skin are closed in standard fashion. An outline of the procedure is shown in Figure 1.

## 3 RESULTS

### 3.1 Study population

The patients' age ranged between 46 and 73 years, the BMI ranged between 20.2 and 27.4 kg/m^2^, and ASA score ranged between 1 and 3. The median (interquartile range, IQR) preoperative PSA level was 7.4 (6.2–9.1) ng/ml. The clinical T stage was cT1 for seven and cT2 for three patients. The detailed demographic and clinical characteristics of the patients are presented in Table 1.

**TABLE 1:** Demographics and clinical characteristics of the cohort

Characteristic	*N* = 10
Median (IQR, range)	
Age, years	70 (62.5–72, 46–73)
BMI, kg/m^2^	23.5 (21.3–24.3, 20.2–27.4)
ASA score	2 (2–2, 1–3)
CCI	5 (4–5, 2–7)
SHIM score	10 (6–14, 1–24)
Prostate volume, ml	25.2 (20–33.3, 15–47.2)
PSA level, ng/ml	7.4 (6.2–9.1, 3.1–13.9)
*N*	
ISUP grade group	
1	3
2	4
3	3
Clinical T stage	
cT1c	7
cT2a/b/c	3
cT3/cT4	0

Abbreviations: ASA, American Society of Anesthesiology; BMI, body mass index; CCI, Charlson Comorbidity Index; IQR, interquartile range; ISUP, International Society of Urological Pathology; SHIM, Sexual Health Inventory for Men.

### 3.2 Surgical outcomes

**FIGURE 2:**
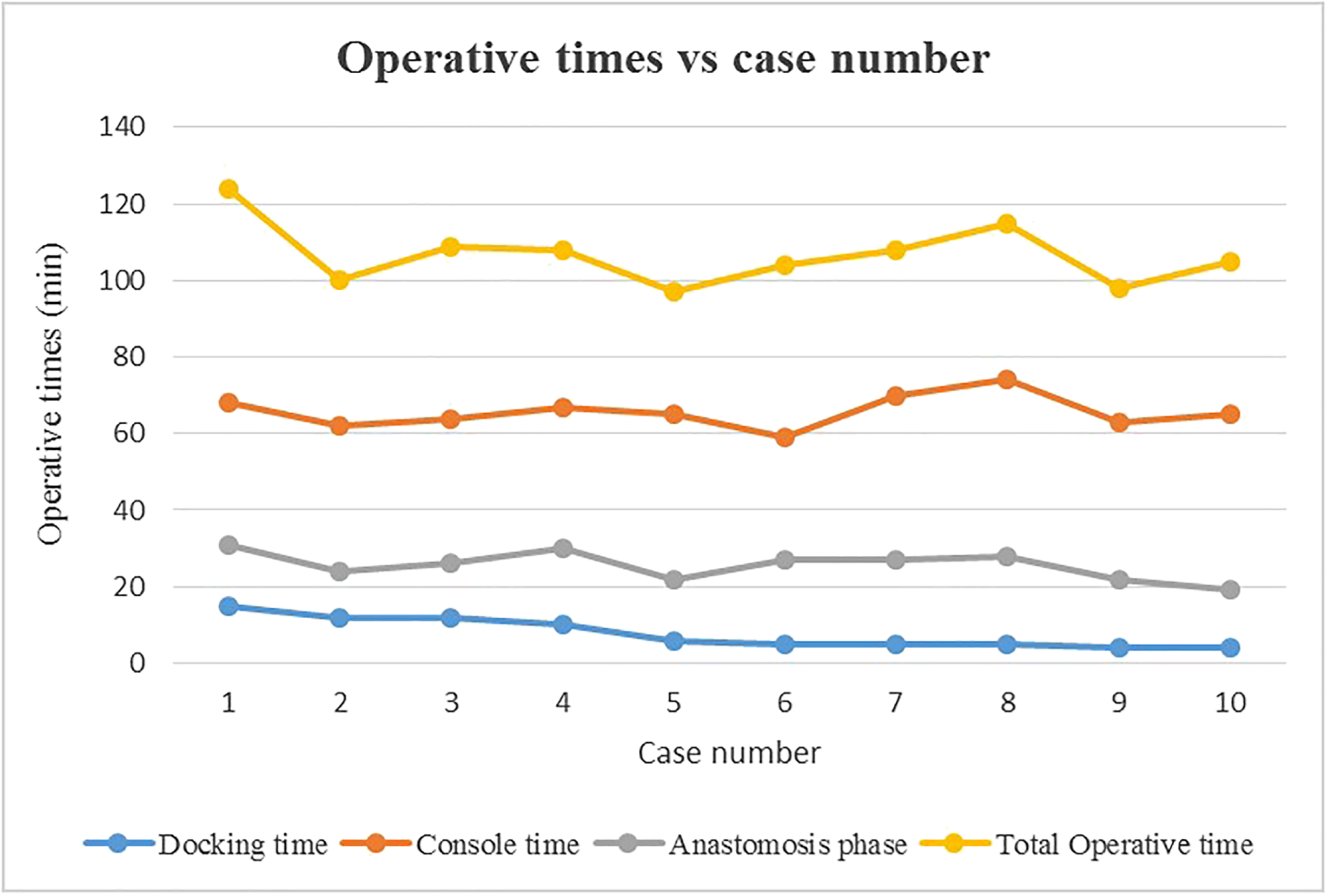
Operative times versus case number

All procedures were successfully performed by the same surgeon and surgical team without any intraoperative complication, adding of extra ports or conversion to multiport or anterior approach. The operative time, docking time, console time, and urethrovesical anastomosis time ranged from 97 to 124, 4 to 15, 59 to 74, and 19 to 31 min, respectively (Figure 2). The EBL ranged from 20 to 200 ml. Postoperatively, one patient had ileus, which resolved spontaneously (Clavien–Dindo grade I) and no major complication occurred. The hospitalization time and catheterization time ranged from 3 to 6 and 6 to 9 days, respectively (Table 2).

**TABLE 2:** Perioperative and postoperative outcomes

Outcome	*N* = 10
Median (IQR, range)	
Total operative time, min	106 (101–109, 97–124)
Console time, min	65 (63–68, 59–74)
Docking time, min	6 (5–11, 4–15)
Urethrovesical anastomosis time, min	26 (22–28, 19–31)
EBL, ml	125 (50–150, 10–200)
Hospital stay, days	3 (3–4, 3–6)
Catheterization time, days	8 (7–9, 6–9)
Clavien–Dindo complications	1 (grade I)
*N*	
Pathological ISUP grade	
1	0
2	5
3	4
4	1
Pathological T stage	
pT2	6
pT3a	3
pT3b	1
Positive surgical margins	5

Abbreviations: EBL, estimated blood loss; IQR, interquartile range; ISUP, International Society of Urological Pathology.

### 3.3 Early oncological and continence outcomes

Pathological examination of the specimen revealed pT2 disease in six patients and pT3 disease in four. Surgical margins were found positive in five patients. Postoperatively, at 1 month nine patients had undetectable levels of PSA and at 3 months none of them met the criteria of BCR. Regarding continence, seven patients were continent immediately after catheter removal. At 3 months after surgery all patients were continent, seven of them were completely dry, and three of them were using a safety liner.

## 4 DISCUSSION

In the present study, we demonstrate our technique for pure SP retzius‐sparing RARP with the da Vinci SP system. Our results indicate that pure SP retsius‐sparing surgery appears to be a feasible and safe approach to performing a radical prostatectomy. In our initial series of 10 consecutive cases we were able to perform successfully this approach without the need for conversion to the anterior approach or extra port insertion during the surgery. Furthermore, only one minor complication (ileus—grade I) was noted in our cohort and EBL was relatively low (<200 ml) for all cases.

Until today, various groups have reported the use of the da Vinci SP system in radical prostatectomies through different approaches,^13^, ^14^, ^15^, ^16^ although prior reports on retzius‐sparing RARP are sparse. Preclinically, Ng et al.^17^ demonstrated this technique in a human cadaver. In a clinical setting, Agarwal et al.^18^ reported their initial experience with the da Vinci SP system in radical prostatectomies in a cohort of 49 patients including 7 cases of retzius‐sparing approach. However, an additional 12‐mm assistant port was used in the right lower quadrant (SP plus one) and a high rate (three out of seven cases) of conversion to anterior approach was noticed. To the best of our knowledge, our study is the first reporting the feasibility and initial results of pure SP retzius‐sparing RARP.

The da Vinci SP could be easily adopted in retzius‐sparing RARP as is an ideal system in working in narrow spaces. Furthermore, the articulating camera adds the benefit of viewing the surgical field from different angles (0° and 30°), which is useful during posterior dissection and anastomosis. The technical differences between this new system and its multiport predecessors in port placement, docking the robot, instruments, and camera maneuverability did not importantly prolong our operative time, which was consistently below 2 h. An improvement in operative times during the cases was only noticed for the docking time, from 15 min at the initial case to around 5 min after the fifth case. It is worth emphasizing that off‐site training of the surgical team and on‐site guidance during the initial cases by trained members of Intuitive Surgical are of paramount importance for a successful transition from multiport da Vinci systems to the SP system.

**FIGURE 3:**
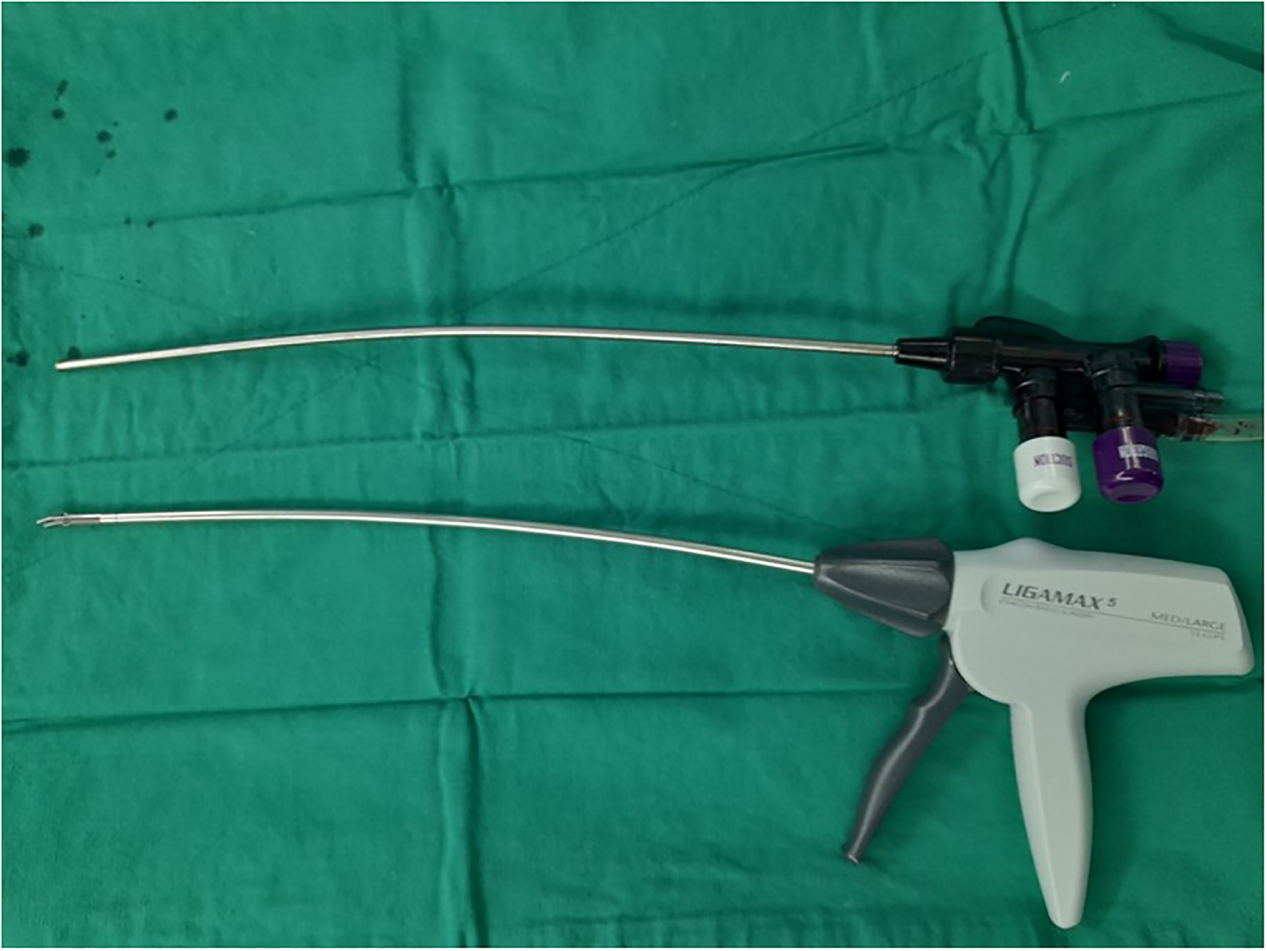
Bent suction tube and clip applier shaft

Despite the da Vinci SP being a purpose‐built single site system, some drawbacks still exist. The system provides 7 degrees of freedom movements; although with a different mechanism, the Endowrist technology is lacking and a novel elbow has introduced. We acknowledge that the surgeon could face difficulties during suturing due to changes in instruments dynamics and lack of wristed movements. From our experience, a significant proportion of the console time was spent during the anastomosis phase. Moreover, with the pure SP technique, the working space of the assistant is quite limited. The surgeon should perform more tasks, and the coordination between the two should be perfect to avoid instrument clashing, mainly with the camera which is placed at 6 o'clock position for our approach. A trick to widen the areas of access for the assistant is to slightly bend the disposable suction tube and the clip applier shaft (Figure 3).

A high rate (5 out of 10 patients) of positive surgical margins, including focal and nonfocal, was identified in final pathology. Nevertheless, four patients of our cohort proved to have extraprostatic disease (pT3a/b) without any preoperative evidence in mp‐MRI. Similar rates of positive surgical margins were found to the initial cases of extraperitoneal SP RARP by the Kaouk's team.^16^ Surgeons interested in adopting our technique should carefully select their initial patients to avoid high rates of positive surgical margins during the learning curve.

We observed excellent continence outcomes in our cohort; seven patients were continent immediately after catheter removal, and all patients were continent at 3 months. In our previous series of multiport retzius‐sparing RARP, the continence recovery rate at 3 months was 87.8%.^19^ Agarwal et al. also reported excellent continence results, where all patients of their cohort who underwent SP retzius‐sparing RARP were continent within 1 week of catheter removal.^18^ It is our impression that the smaller instruments (6 mm) and the less traction forces applied by the SP robot could have a positive impact on functional results. A recent comparative study between the da Vinci SP and Xi robots for patients undergoing RARP with the anterior approach showed also better continence results for the SP robot, suggesting that difference in continence rates at 45 days between the SP and Xi groups were 11% (95% CI −5.6% to 28%).^20^

Our study was not devoid of limitations. We included a small cohort of patients with the primary outcome of our study to be the feasibility and description of the technique. The short‐term follow‐up is another limitation. The long‐term oncologic outcomes and the benefits of the SP over the multiport approach in terms of cosmesis, postoperative pain, and patient recuperation need further research. Lastly, all the procedures in this study were performed by an experienced robotic surgeon in a tertiary hospital and the results maybe are not applicable to novice surgeons.

In conclusion, pure SP retzius‐sparing RARP is a feasible approach for the surgical treatment of prostate cancer. The da Vinci SP system could be easily adopted by an experienced surgical team in this skill‐intensive procedure, in acceptable surgical times, and without compromising patient safety. The advantages and the long‐term oncologic and functional outcomes of this approach should be further evaluated by future studies.

## CONFLICT OF INTEREST

All the authors have no conflicts of interest to declare.

## FINANCIAL INFORMATION

None.

## AUTHOR CONTRIBUTION

Conception and design: Periklis Koukourikis, Ali Alqahtani, Woong Kyu Han and Koon Ho Rha. Data acquisition: Periklis Koukourikis and Ali Alqahtani. Data analysis and interpretation: Periklis Koukourikis and Ali Alqahtani. Statistical analysis: Periklis Koukourikis. Drafting the manuscript: Periklis Koukourikis. Critical revision of the manuscript for scientific and factual content: Woong Kyu Han and Koon Ho Rha. Supervision: Woong Kyu Han and Koon Ho Rha. Approval of the final manuscript: all authors.
